# Discovery of Genes Related to Witches Broom Disease in *Paulownia tomentosa × Paulownia fortunei* by a *De Novo* Assembled Transcriptome

**DOI:** 10.1371/journal.pone.0080238

**Published:** 2013-11-21

**Authors:** Rongning Liu, Yanpeng Dong, Guoqiang Fan, Zhenli Zhao, Minjie Deng, Xibing Cao, Suyan Niu

**Affiliations:** 1 Institute of Paulownia, Henan Agricultural University, Zhengzhou, Henan, P. R. China; 2 College of Forestry, Henan Agricultural University, Zhengzhou, Henan, P. R. China; University of Innsbruck, Austria

## Abstract

In spite of its economic importance, very little molecular genetics and genomic research has been targeted at the family *Paulownia* spp. The little genetic information on this plant is a big obstacle to studying the mechanisms of its ability to resist Paulownia Witches’ Broom (PaWB) disease. Analysis of the *Paulownia* transcriptome and its expression profile data are essential to extending the genetic resources on this species, thus will greatly improves our studies on *Paulownia*. In the current study, we performed the *de novo* assembly of a transcriptome on *P. tomentosa* × *P. fortunei* using the short-read sequencing technology (Illumina). 203,664 unigenes with a mean length of 1,328 bp was obtained. Of these unigenes, 32,976 (30% of all unigenes) containing complete structures were chosen. Eukaryotic clusters of orthologous groups, gene orthology, and the Kyoto Encyclopedia of Genes and Genomes annotations were performed of these unigenes. Genes related to PaWB disease resistance were analyzed in detail. To our knowledge, this is the first study to elucidate the genetic makeup of *Paulownia*. This transcriptome provides a quick way to understanding *Paulownia*, increases the number of gene sequences available for further functional genomics studies and provides clues to the identification of potential PaWB disease resistance genes. This study has provided a comprehensive insight into gene expression profiles at different states, which facilitates the study of each gene’s roles in the developmental process and in PaWB disease resistance.

## Introduction

The *Paulownia* plant is a fast-growing deciduous hardwood species that belongs to the monogeneric family Paulowniaceae, which is sometimes included in the family Scrophulariaceae. This species is native to China, where it has been cultivated for over 2,000 years. *Paulownia* spp. have a very wide distribution in China and are intercropped on 2.5 million ha of farmland [1], [2]. It has also been introduced successfully into many other countries, like Japan, Korea, and India, in Asia, Europe, America, and more recently Australia [3], [4]. The wood of *Paulownia* is lightweight, strong, soft, straight-grained, and mostly knot-free with a satiny luster [5]. It has a wide variety of commercial uses ranging from log homes, non-structural building uses, and paper making to furniture and musical instrument production [4], [6], [7]. Because of its well-known features, such as rapid growth and high biomass production, this species could be an excellent source of feedstock for the biofuel industry, although its potential has not been investigated. Besides its extensive use as a forestry tree, *Paulownia* can be used for pollution control (both air and soil), land reclamation and as a fast-growing ornamental tree providing both shade and very attractive flowers [8]–[11].

Paulownia Witches’ Broom (PaWB) disease is the major lethal disease of Paulownia trees nearly all over the world [12], [13]. Infected Paulownia trees are characterized by the proliferation of branches with very small yellowish leaves, the witches’ brooms, which is followed by branch dieback. PaWB is caused by phytoplasmas belonging to the Aster Yellows group ‘*Candidatus* Phytoplasma asteri’ (IRPCM Phytoplasma Taxonomy Group 2004). PaWB phytoplasma is reportedly transmitted by the stinkbug *Halyomorpha mista* Uhler and *H. halys* Stål. During the last thirty years, a lot of research has been carried out into the ecology and biology characteristics of PaWB, but the mechanisms behind its molecular regulation remain poorly understood.

Even with the current achievements in the molecular identification of PaWB, a comprehensive view of this disease has yet to form, largely because of the lack of Paulownia genomic information. As of May 1^st^, 2013, there had been only 124 Paulownia nucleotide sequences and 130 protein sequences submitted in the NCBI database. These data are far from enough. Most of the important genes involving in PaWB disease resistance are still unknown. Traditional biochemical methods cannot fully characterize gene sequences. PCR combined with RACE is usually a time-consuming, sometimes inefficient process [14]. Next-generation high-throughput DNA sequencing techniques have offered hopes to quickly and efficiently obtain enormous quantities of genetic information [15]. In recent years, RNA sequencing has revolutionized the exploration of gene expression. The transcriptome analysis platform Illumina HiSeq 2000 has been used in research of animals, plants, fungi and bacteria [16]–[18]. In plants, the Illumina transcriptome analysis has been a precise and reliable way to study genomic characteristics under abiotic and biotic stresses [14], [19]–[24]. This technique has yet been applied to Paulownia. Thus, transcriptome analysis on Paulownia will provide large information of this species at the molecular level.

An experiment was conducted to study the effects of methyl methane sulfonate (MMS) on the morphological changes of *P. tomentosa* × *P. fortunei* seedlings with PaWB. The results demonstrated that the seedlings treated with 15 mg·L^−1^ of MMS for 3 h retained the disease symptoms. Those treated with 15 mg·L^−1^ for 5 h or with more than 30 mg·L^−1^ for longer than 3 h were healthy. However, the phytoplasma was detected in the seedlings treated with 15 mg·L^−1^ for 5 h by nested PCR [25]. In the current study, we applied short-read sequencing technology (Illumina) and *de novo* transcriptome analysis. We constructed a sufficiently large library covering four samples of *P. tomentosa* × *P. fortunei*: PTF (healthy), PTFI (phytoplasma infected), PTFI-15 (phytoplasma infected and 15 mg·L^−1^ MMS treated) and PTFI-30 (phytoplasma infected and 30 mg· L^−1^ MMS treated). 203,644 unigenes were assembled by Trinity from nearly 336 million reads of 29 billion nucleotides (nt). Of all the unigenes with complete structures, 32,976 (30%) matched known proteins in the NCBI database with a BLAST search. Those matches included a number of genes involving in PaWB disease resistance. The genes that are potentially involved in PaWB disease resistance were identified. These transcriptome data, which are assembled and annotated, provide a great deal of genomic resources available for researchers studying Paulownia, allow for a better understanding of the molecular processes of PaWB resistance, and may provide a shortcut to identifying candidate genes potentially related to this important process.

## Materials and Methods

### Materials

All of the biological materials were from the Institute of Paulownia, Henan Agricultural University, Zhengzhou, Henan Province, China. The tissue culture seedlings of *Paulownia tomentosa* × *Paulownia fortunei*, PTF(three individuals) and PTFI (nine individuals) were cultured for 30 days before being clipped from the roots. PTFIs were transferred into 100 ml triangular flasks containing 1/2 MS culture mediums [25] containing 25 mg·L^−1^ sucrose and 8 mg·L^−1^ agar (Sangon, Shanghai, China) with 0, 15, or 30 mg·L^−1^ MMS, respectively. PTF was transferred into 100 ml triangular flasks containing 1/2 MS medium without MMS as the control. At least three parallel samples were prepared for each condition. All samples were first cultured at 20°C in the dark for 5 days. After that, they were cultured under 25±2°C and 130 µmol ·m ^−2^ s^−1^ intensity light for 16 h every day.

### RNA Isolation

For each treatment, approximately 8 mg of leaves were homogenized in liquid nitrogen with a pestle. Total RNA was extracted from the cells using TRIzol reagent (Invitrogen, Carlsbad, CA, USA), followed by RNA purification using the RNeasy MiniElute Cleanup Kit (Qiagen, Dusseldorf, Germany), according to the manufacturer’s protocol. RNA was quantified by measuring the absorbance at 260 nm using a NanoVue UV-Vis spectrophotometer (GE Healthcare Bio-Science, Uppsala, Sweden). The purity of all RNA samples was assessed at an absorbance ratio of OD260/280 and OD260/230, and the integrity of RNA was confirmed by 1% agarose gel electrophoresis.

### Construction of the cDNA Library and Illumina Sequencing for Transcriptome Analysis

For mRNA library construction and deep sequencing, RNA samples were prepared using the TruSeq RNA Sample Preparation Kit according to the manufacturer’s protocol.

Briefly, 4 µg total RNA (a mixture of RNA from four samples) was used to construct a cDNA library. Poly-A containing mRNA molecules were purified from total RNA using poly-T oligo-attached magnetic beads in two rounds of purification. During the second elution of the poly-A RNA, the RNA was fragmented into small pieces using divalent cations at 95°C. The cleaved RNA fragments were reverse transcribed into first strand cDNA using random hexamer primers. Second strand cDNA was synthesized using RNaseH and DNA polymerase I. The cDNA fragments were end-repair processed to convert the overhangs into blunt ends, using an End Repair (ERP) mix. The 3′ to 5′ exonuclease activity of this mix removes the 3′ overhangs and the polymerase activity fills in the 5′ overhangs. A single ‘A’ nucleotide was then added to the 3′ ends of the blunted fragments to prevent them from ligating to one another during the adapter ligation reaction. A corresponding single ‘T’ nucleotide on the 3′ end of the adapter provides a complementary overhang for ligating the adapter to the fragment. This strategy ensures a low rate of chimera (concatenated template) formation. The multiple indexing adapters were ligated to the ends of the dscDNA, preparing them for hybridization to a flow cell. PCR was used to selectively enrich the DNA fragments that had adapter molecules on both ends and to amplify the DNA in the library. The number of PCR cycles was minimized to avoid skewing the representation of the library. A gel purification procedure was performed to select the fragments sized from 250 to 350 bp to produce a library for cluster generation and sequencing. The library was qualified by an Agilent 2100 bioanalyzer and quantified by Qubit and PCR. The cluster formation and sequencing on the GA*_IIX_* platform were performed following the manufacturer’s standard cBot and sequencing protocols. For the multiplex sequencing, a 100 cycles of single read 1 were used to sequence the RNA, followed by seven cycles of index identification and 100 cycles of single read 2. Primary data analysis and base calling were performed by the Illumina instrument’s software. Advanced data analysis was based on the strategy described in the *Bioinformatics analysis* section. The data used in this publication have been deposited in the NIH Short Read Archive database (http://www.ncbi. nlm.nih.gov/sra) and are accessible through SRA accession number SRP028911 (Alias: PRJNA215796).

### Bioinformatics Analysis

The raw reads still contain adapters, unknown or low quality bases. These contaminants were filtered out with SolexaQA’s DynamicTrim.pl (http://solexaqa.sourceforge.net/) to trim remaining sequences based on quality so as to produce clean reads. The high quality reads were analyzed by Trinity to construct unique consensus sequences. The trimmed Solexa transcriptome reads were mapped to the unique consensus sequences using SOAP2 [26]. Sequences with the least numbers of Ns and that could not be extended on either end were defined as unigenes. Unigenes from each sample’s assembly can be taken into further process of sequence splicing and redundancy removing with sequence clustering software to generate the longest possible non-redundant unigenes. The unigenes were then screened against the NCBI Non-redundant nucleotide database (NT, as of May 22^nd^, 2011) and Non-redundant protein database (NR, as of May 22^nd^, 2011) using BLASTN [27] and BLASTX [27], respectively, with the same E-value cutoffs ≤10^−5^. Unigenes were identified by sequence similarity comparisons against the SWISS-PROT (SWISS-PROT downloaded from European Bioinformatics Institute by May 22^nd^, 2011) [28] with BLAST [27] at E values ≤10^−10^. Unigenes were assigned functional annotation by sequence similarity comparisons against the Eukaryotic clusters of orthologous groups (KOG) database [29], [30] with BLAST at E values ≤10^−10^. Unigenes were then classified corresponding to different functional classes and compared with the Kyoto Encyclopedia of Genes and Genomes (KEGG) database [31] using BLASTX [27] at E values ≤10^−10^. KO information were retrieved from blast results and then established pathway associations between the unigene and the database. InterPro domains [32] were annotated by InterProScan [33] Release 27.0 and functional assignments were designated by gene orthology (GO) [34]. WEGO [35] was used to perform GO classifications and draw a GO tree.

### Comparison of Gene Expression Profiles among the Different Samples

**Figure 1:**
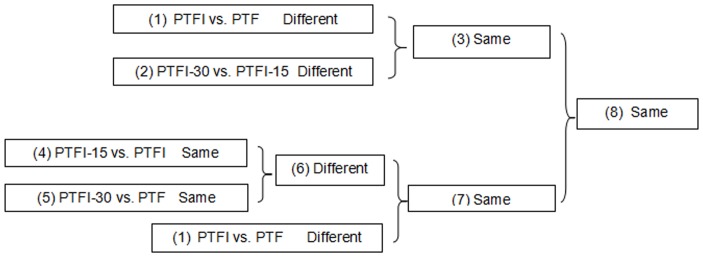
Comparison schemes of the four samples. PTF represents the healthy wild-type sample of *P. tomentosa* × *P. fortunei,* PTFI represents the sample of phytoplasma infected PTF. PTFI-15 represents the sample of 15 mg·L^−1^ MMS treated PTFI. PTFI-30 represents the sample of 30 mg·L^−1^ MMS treated PTFI.

The four samples were evaluated in several pairwise comparisons (Figure 1): (1) Differentially expressed genes were chosen during the PTFI vs. PTF comparison to screen for genes involved in PaWB disease and other factors, like developmental differences of samples (DDS) and the influences of the methylating agent MMS (IMM). (2) Differentially expressed genes were chosen during the PTFI-30 vs. PTFI-15 comparison to screen for genes involved in PaWB disease and other factors, like DDS. (3) The same genes from 1 and 2 were compared with genes involved in PaWB disease and DDS. (4) The same expressed genes were chosen during the PTFI-15 vs. PTFI comparisons to identify genes involved in DDS and IMM. (5) The same expressed genes were chosen during the PTFI-30 vs. PTF comparisons to identify genes related to DDS only. (6) The different genes from comparison 4 and 5 were related to IMM only. (7) During comparison 1, DDS and IMM were involved, but different genes were chosen from the background of comparison 6. Thus, the same genes from comparison 5 and 1 were compared as genes with IMM only. (8) The same genes from comparisons 3 and 7 were related to PaWB only without DDS and IMM considered.

### Analysis of Genes Related to PaWB Disease Resistance and Target Enzymes

Differentially expressed genes were identified using Cufflinks (http://cufflinks.cbcb.umd.edu/). The calculation of unigene expression levels was performed by the FPKM value (Fragments Per kb per Million fragments) [36]. We developed a rigorous algorithm to identify differentially expressed genes between two samples by referring to Audic et al [37]. To identify differentially expressed genes in two samples, the false discovery rate (FDR) method was used to determine the threshold of the P-value in multiple tests [38]. The significance of differences in gene expression was judged using a threshold FDR <0.001 and an absolute value of log_2_ Ratio >1.5. Differentially expressed genes were then subjected to GO functional analysis and KEGG pathway analysis.

### Quantitative Real-time PCR (qRT-PCR) Analysis of Differentially Expressed Genes

The RNA samples from the leaves of the PTF, PTFI, PTFI-15, and PTFI-30 samples were extracted with Trizol (Sangon, Shanghai, China). The RNA was then precipitated with isopropanol. Purified and concentrated RNA was denatured and first-strand cDNAs for all the samples were synthesized using a PrimeScript RT reagent Kit (Takara, Dalian, China). Potential genes related to PaWB disease resistance were chosen. The primers were designed with Beacon Designer, version 7.7 (Premier Biosoft International, Ltd., Palo Alto, CA, USA). The cDNAs were then amplified in a Bio-Rad CFX96TM Real-Time System (Bio-Rad, Hercules, CA, USA) with SYBR Premix Ex Taq TM II (Takara, Dalian, China). The following PCR parameters were used: 50°C for 2 min, 95°C for 30 s followed by 40 cycles of 94°C for 15 s and 60°C for 1 min. Three replicates were analyzed for each gene. The average threshold cycle (Ct) was normalized and the relative expression changes were calculated using the 2^−△△Ct^ method. The 18S rRNA of *Paulownia* was chosen as an internal reference gene for normalization. All the primers used for the qRT-PCR analysis, potential gene functions, and amplicon sizes are shown in Table 1.

**Table 1:** Primers of quantitative RT-PCR analysis of candidate defence genes. -f represents forward primers and -r represents reverse primers.

Potential gene function	Size (bp)	Primer	Sequence
purple acid phosphatase 23	1224	m. 16215-f	GCCTGAATCAATGTCAATACC
		m. 16215-r	CTGCTGCTACTACAATTATGC
WRKY transcription factor 20	1098	m. 28831-f	GCAGGTTCTTGACATCATTAG
		m. 28831-r	AGGAAGAACAGGAACAAGAGAAC
hexose carrier	696	m. 34711-f	GTCTCATTCTATGCTCCACTATTG
		m. 34711-r	ACCCTTCTGCCTACTTTATCTAC
heat shock protein 70	1224	m. 35228-f	CCACCAGCGGACAAGAAG
		m. 35228-r	GCCATATCAGCACCACCAG
glutathione S-transferase	438	m. 31344-f	TGGCTGGATTGGAATTACTG
		m. 31344-r	CGTACATGGTCGGATAAGG
pyruvate kinase	1398	m. 50337-f	CAGTATGGCTTGAGGTTATAG
		m. 50337-r	AAGGAAGTCTATGTTGTTGC

## Results

### Sequencing and Sequence Assembly

A library of four different samples (PTF, PTFI, PTFI-15, PTFI-30) was constructed using Illumina (paired-end) sequencing technology in a single run that generated 471,738,404 total reads with an average length of 100 bp. The 335,278,558 high quality reads with 28,687,625,164 nucleotides (nt), an average length of 85 bp and a Q20 percentage of 95%, were chosen for assembly (Table 2). These short reads were assembled *de novo* into 203,664 unigenes using the Trinity programs. They ranged in length from 201 to 17,086 bp, with a mean of 1,328 bp. Out of these unigenes, 134,062 unigenes (65.83%) were longer than 500 bp and 95,649 (46.96%) were larger than 1,000 bp. The length distribution of these unigenes is shown in Figure S1. The gap distribution of the unigenes was analyzed to assess the data quality. The unigenes contained no gaps, thus demonstrating the high quality of the assembly.

**Table 2:** Summary of the transcriptome.

Items	Numbers
Total reads	235,869,202
Total clean reads	167,639,279
Total nucleotides (nt)	28,687,625,164
Total Average length (bp)	85
Total number of unigenes	203,664
Mean unigene length (bp)	1,328

### Annotation of Predicted Proteins

To obtain and validate sequence-based annotations for all assembled unigenes, ORF (open reading frame) of these unigene sequences were predicted using the Trinity program. There were 32,976 unigenes with whole ORF structures that were chosen. The total length of these unigenes was 83,011,675 bp, which comprised 30% of all the unigenes. The length of these unigenes ranged from 376 bp to 12,339 bp with a mean length of 2,517 bp. The length distribution of these unigenes is shown in Figure S2A. A total of 36,061,416 bp of CDS ranging in length from 300 bp to 11,238 bp and having a mean length of 1094 bp were found. The length distribution of these CDSs is shown in Figure S2B. The annotation of these ORFs was prepared by searching the non-redundant (nr) NCBI protein, Swiss-Prot, KEGG, KOG and Pfam data bases using BLASTP with a cutoff E-value of 10^−5^. A total of 31,057 distinct sequences (94.2% of ORFs) matched known genes in the nr database, 24,155 sequences (73% of ORFs) matched known genes in Swiss-Prot, 9,015 sequences (27% of ORFs) matched known genes in KEGG, 14,069 sequences (43% of ORFs) matched known genes in Pfam, and 19,593 sequences (59% of ORFs) matched known genes in KOG (Table 3).

**Table 3:** Annotation of the unigenes.

Database name	Numbers of unigenes	Percentage(100%)
Nr	31057	94
Swiss-Prot	24155	73
KEGG	9015	27
Pfam	14069	43
KOG	19593	59

### Unigene Functional Annotations

**Figure 2:**
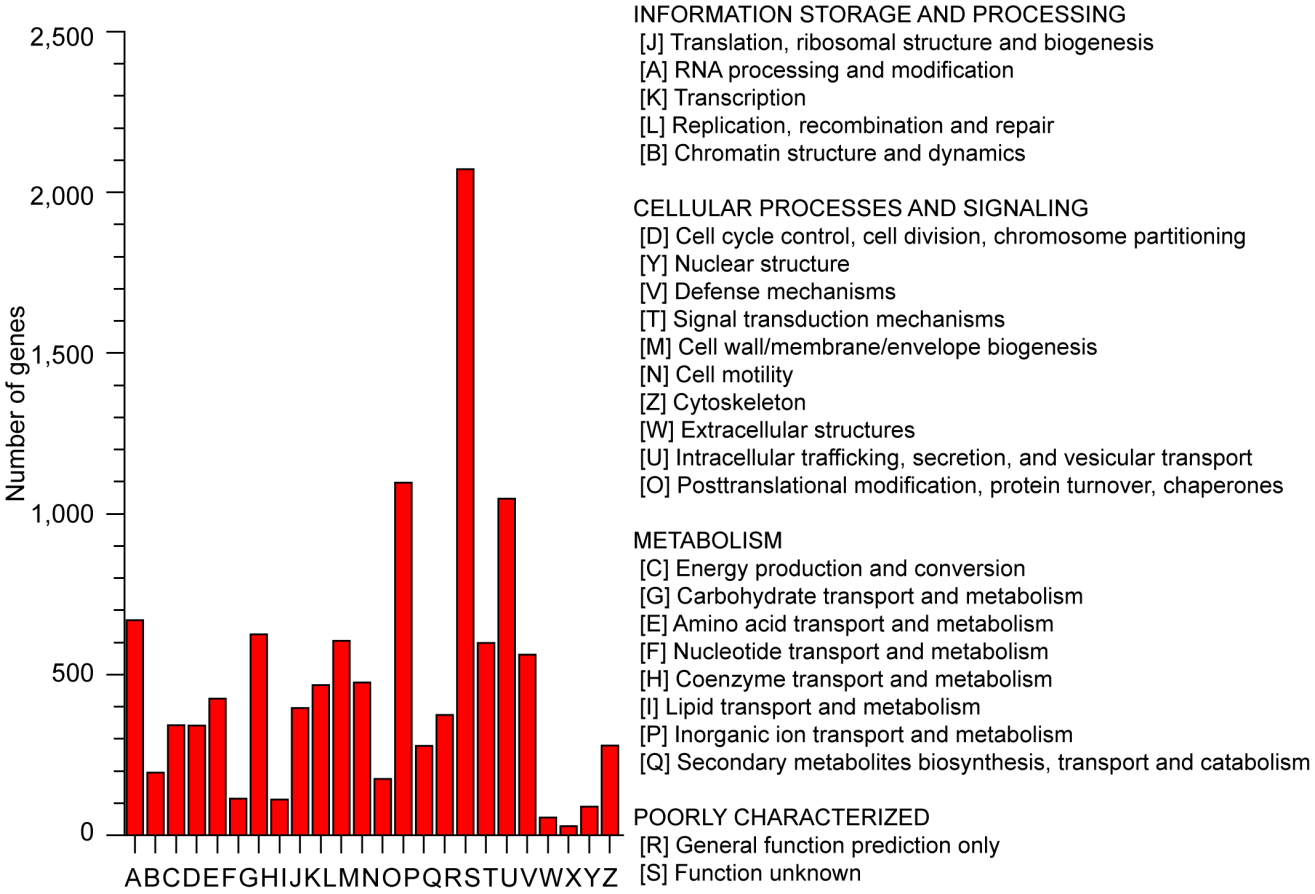
Classification of Eukaryotic clusters of orthologous groups (KOG) for the transcriptome of *P. tomentosa* × *P. fortunei*. 19,593 unigenes (59% of the total ORFs) were annotated and divided into 25 specific categories.

**Figure 3:**
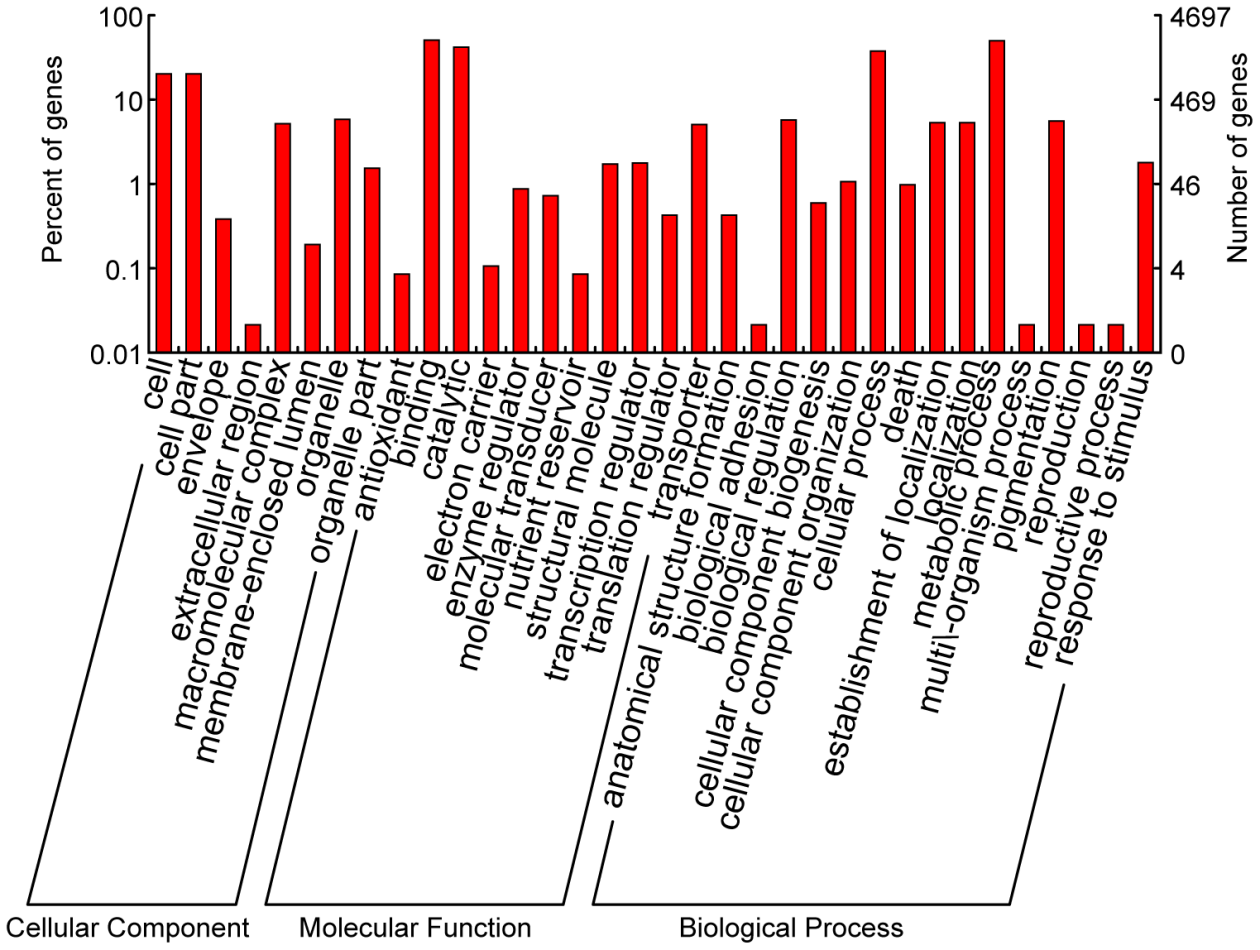
Classification of the gene ontology (GO) for the transcriptome of *P. tomentosa* × *P. fortunei*. Unigenes were categorized into 35 function groups.

Assignments of KOGs were used to predict and classify possible functions of the unigenes. Based on sequence homology, 19,593 unigenes (59% of total ORFs) were annotated and then divided into 25 specific categories (Figure 2). The general function category, which contained 4,020 unigenes (12.19%), was the largest, followed by posttranslational modification, protein turnover and chaperones (2,187, 6.63%), signal transduction mechanisms (2,063, 6.26%), function unknown (1,483, 4.50%), carbohydrate transport and metabolism (1,149, 3.48%) and transcription (1133, 3.44%). Only four unigenes (0.01%) belonged to the Myelin proteolipid protein, which was the smallest group. For GO analysis, unigenes were divided into three ontologies: molecular function, cellular component, and biological process. We categorized these unigenes into 35 function groups. Binding and metabolic process were the two largest groups, containing 7,055 and 5,706 unigenes, respectively. Only four unigenes were predicted to act in biological adhesion (Figure 3).

### Unigene Metabolic Pathway Analysis

The unigene metabolic pathway analysis was conducted using the KEGG annotation system. We mapped 9,015 unigenes to 289KEGG pathways. Metabolic pathways contained 2,555 unigenes and were significantly larger than the other pathways, such as biosynthesis of secondary metabolites (1,185 unigenes), purine metabolism (323 unigenes), and RNA transport (232 unigenes).

### Function Annotation of the Differentially Expressed Genes

**Figure 4:**
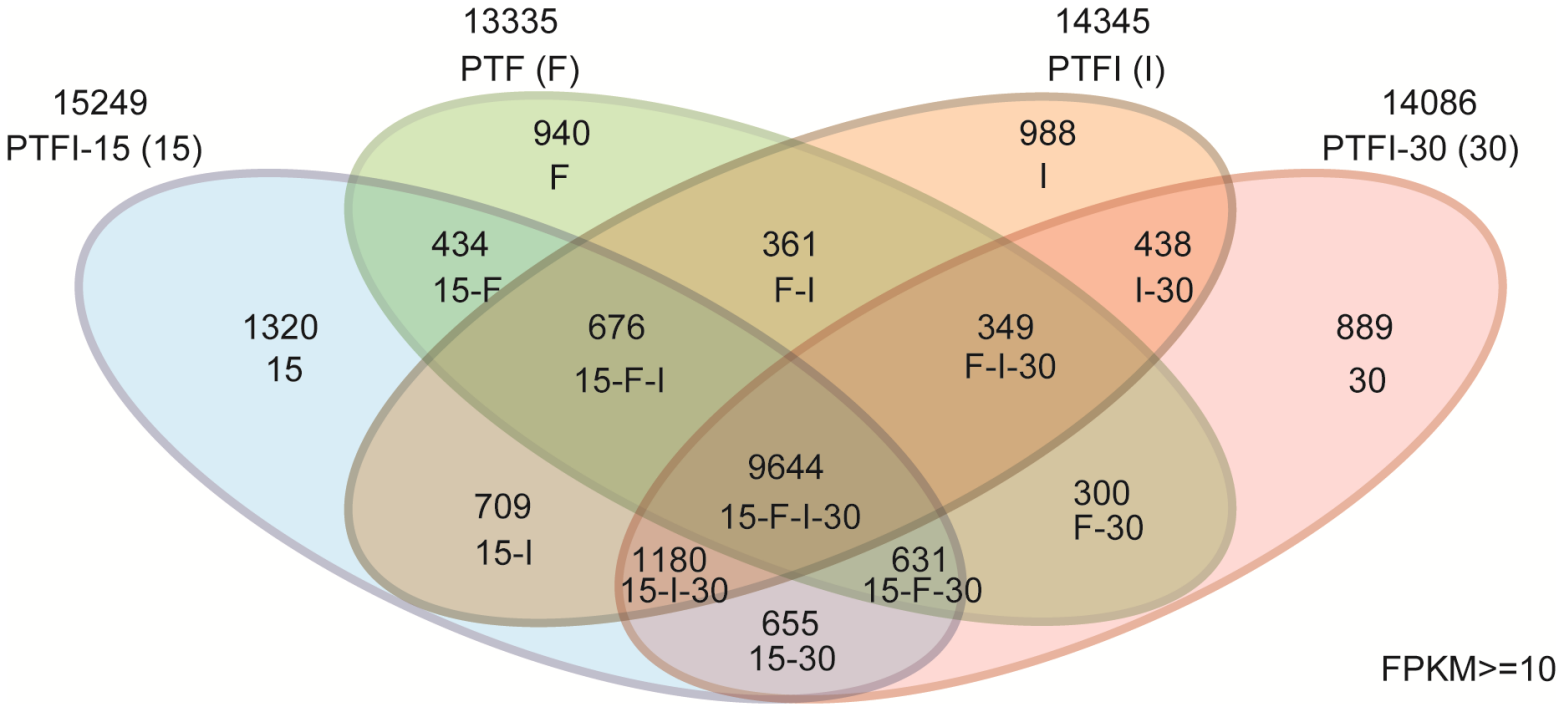
Comparative summary of *P. tomentosa* × *P. fortunei* transcriptomic sequences with the protein sequences of different samples. PTF, healthy. PTFI, phytoplasma infected. PTFI-15, phytoplasma infected and 15 mg·L^−1^ MMS treated. PTFI-30, phytoplasma infected and 30 mg·L^−1^ MMS treated.

A Venn diagram was generated to show the comparative summary of *P. tomentosa × P. fortunei* transcriptomic sequences with the protein sequences of PTF,PTFI,PTFI-15 and PTFI-30 (Figure 4). With the FPKM method described in the Methods section, a rigorous algorithm was developed to identify differentially expressed genes between two samples by referring to Audic et al [37]. The FDR method was used to determine the threshold of the P-value in multiple tests [38]. Using a threshold FDR <0.001 and an absolute value of log_2_Ratio >1.5, genes with significant differences in expression levels were identified (Table S1). The results suggested that the expression levels of 74 genes were significantly different. Assignments of KOGs were used to predict and classify the possible functions of the differentially expressed unigenes. Based on sequence homology, 40 unigenes were annotated and then divided into 16 specific categories (Figure S3). The general function category, which contained nine unigenes, was the largest, followed by posttranslational modification, protein turnover and chaperones (five unigenes). Differentially expressed genes were characterized into three groups based on the GO classification: biological process, cellular component, and molecular function. The results of the comparisons showed 63 unigenes were catalogued into 16 function groups. Binding and catalytic activity were the two largest groups, containing 16 and eight unigenes, respectively (Figure S4). In the pathway analysis, the most differentially expressed genes were involved in metabolic pathways and biosynthesis of secondary metabolites (Figure S5).

### Confirmation of Six Differentially Expressed Genes by qRT-PCR

**Figure 5:**
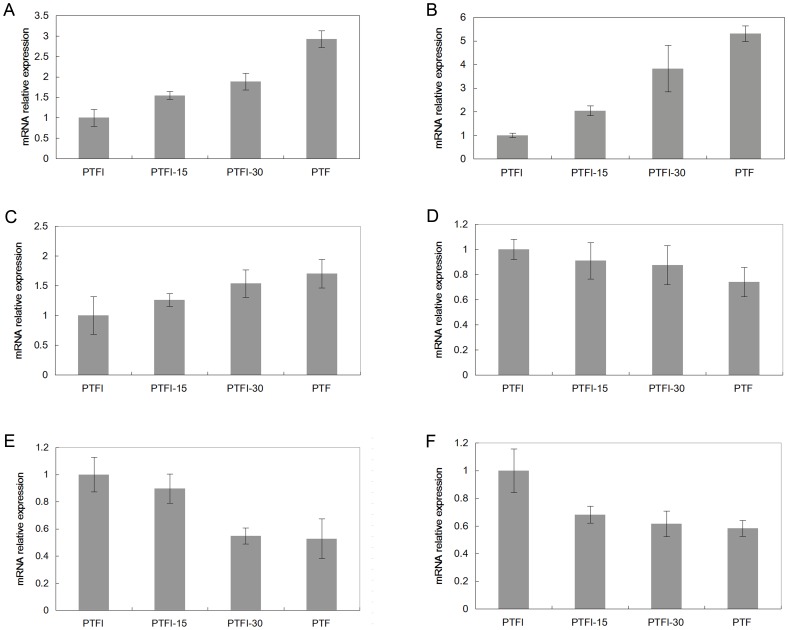
Quantitative RT-PCR analysis of candidate defence genes. PTF, healthy. PTFI, phytoplasma infected. PTFI-15, phytoplasma infected and 15 mg·L^−1^ MMS treated. PTFI-30, phytoplasma infected and 30 mg·L^−1^ MMS treated. (A) mRNA levels for a purple acid phosphatase. (B) mRNA levels for a WRKY transcription factor. (C) mRNA levels for a hexose carrier. (D) mRNA levels for a heat shock protein 70. (E) mRNA levels for a glutathione S-transferase. (F) mRNA levels for a pyruvate kinase. 18S rRNA was used as the internal reference gene. Standard error of the mean for three technical replicates is represented by the error bars.

To confirm the reliability of Solexa/Illumina sequencing technology, six candidate genes were selected for qRT-PCR assays. Compared to the expression levels in the healthy samples, the transcription levels of a purple acid phosphatase, a WRKY transcription factor and a hexose carrier decreased significantly in the phytoplasma infected ones. Under MMS treatment, the expression levels of these genes were found to be higher than those in the healthy samples but lower than those in the phytoplasma infected ones. Also, they increased with the increase of the MMS concentrations. However, the transcription level of a heat shock protein 70, a glutathione S-transferase and a pyruvate kinase were found to be opposite. The results indicated that expressions of all six genes were consistent between the qRT-PCR and the transcriptome analyses (Figure 5), confirming the validity of the sequencing method. Furthermore, the functions of these genes involving in PaWB phytoplasma response were confirmed.

## Discussion

The molecular mechanisms involved in symptom development and interaction between phytoplasmas and their hosts are largely unknown. Using the RNA-seq technique, we identified a series of plant genes whose expression is altered between healthy and infected plants and between symptomatic and non-symptomatic plants. Some of the differentially expressed fragments did not show any similarity with sequences in the databases, representing potentially novel proteins important in interactions with, and perhaps related to, the specific plant responses to phytoplasmas.

Based on the functions of the differentially expressed genes, a model is proposed to interpret the role of these genes in the Paulownia-phytoplasma compatible interactions that cause symptom expression and plant responses to phytoplasma infection.

Transient changes in the ion (Ca^2+^, K^+^, H^+^) permeability of the plasma membrane appear to be a common early event in stress signaling [39]. This is generally followed by the synthesis and release of secondary messengers, such as reactive oxygen species (ROS) [40]. ROS (e.g. H_2_O_2_) play a central role in the defense of plants against pathogens [41], [42], and the key enzymes involved in removing ROS are antioxidants, such as cytochrome P450. In the case of our study, a gene encoding a cyclic nucleotide-gated ion channel protein (m. 41108)involved in the plant-pathogen interaction pathway (map 04626) was differentially expressed. Thus, we speculate that like other stresses, the cellular ROS concentration increased after being challenged with phytoplasma, which induced the expression of heat shock protein 70 encoded by m. 35228 and the activation of the 90 kDa heat shock protein ATPase homolog 1 encoded by m. 12337. However, the levels of antioxidants, such as glutathione S-transferase encoded by m. 31344, were likely increased in the compatible interactions (susceptible hosts) by the cell to reduce oxidative damage. In our case, the ROS reduction probably resulted in the suppression of the defense pathways in the susceptible (PTFI) infected host.

The second messengers can initiate a protein phosphorylation cascade [43]; therefore, the differential regulation pattern of purple acid phosphatase 23 encoded by m. 16215 is not unexpected, and this component mediates phytoplasma-induced signal transduction pathways. These signals may activate the expression of genes encoding transcription factors (TFs). The TFs identified in the present study, such asWRKY encoded by m. 28822 and m. 28831, AP2-like encoded by m. 30036, ASG4 encoded by m. 18108, and SR2L encoded by m. 554812, activate the stress-inducible genes during symptom expression and physiological changes stimulated by infection [43], [44]. Hereafter, in the proposed model, the identified genes are classified into four groups based on their roles in the regulation of plant responses to phytoplasma infection: 1) genes related to photosynthesis pathways and carbohydrate metabolism; 2) genes involved in symptom expression; 3) genes involved in the regulation of plant defense mechanisms; and 4) genes involved in secondary metabolism.

### Paulownia Photosynthesis and Carbohydrate Metabolism Genes Responding to Phytoplasma

There is growing evidence that several steps in photosynthesis are repressed during phytoplasma infection and that its general chlorosis is a result of accumulating carbohydrates in source leaves [45]. However, it is not clear if the observed alterations are directly linked to the presence of phytoplasmas or, as proposed by Bertamini *et al*. [46], [47], are non-specific, general responses to the infection. Nevertheless, our results provide new insights on the photosynthesis-associated proteins in phytoplasma-infected plants at the transcriptome level, and thus complement already obtained biochemical data from studies on *C. roseus* infected with ash yellows phytoplasmas, on apple trees infected with phytoplasmas causing apple proliferation and grapevines infected with ‘BN’ phytoplasma [46], [47]. Based on the indirect measurement of the variable fluorescence yield, together with the loss of several thylakoid polypeptides, it has been proposed that in phytoplasma-infected leaves, it is mainly photosystem II (PSII) that is impaired, particularly at the donor site [46], [47]. Our results support this hypothesis. One gene (m.50337) encoding a pyruvate kinase in the carbon fixation pathway of PSII (map00710) was significantly differentially expressed only in the symptomatic part of the plants. This gene was identified, and the differential expression of this gene is probably due to carbohydrate accumulation in the symptomatic leaves [48]. Its synthesis is specifically repressed by free hexoses [49], whose accumulation in leaves is generally associated with phytoplasma infections [50]–[54]. Thus, the hexose carrier protein HEX6 encoded by m. 34711 is found to be differentially expressed. The result is in accord with the study of a ‘Bois noir’ phytoplasma induced reprogramming of the leaf transcriptome in the field grown grapevine. As shown by the microarray analyses, the transcripts of the genes encoding vacuolar invertase and sucrose synthase, which can catalyze sucrose into hexoses were up-regulated upon ‘BN’ phytoplasma infection [55]. Moreover, the expression of the gene coding for the pyruvate kinase was also a key enzyme in the Calvin cycle, which may participate in the process of glycolysis/gluconeogenesis (map00010) and pyruvate metabolism (map 00620). Thus, part of photosynthesis reduction under phytoplasma infection may be due to changes in this gene’s expression. Together, these data suggest a detrimental effect of phytoplasma infection on photosynthetic pathways. These results are consistent with previous reports that environmental stresses inhibit the expression of genes that encode photosynthetic proteins [56]–[58]. The differential expression and degradation of photosynthetic proteins has also been revealed by the proteomic analysis of the response of mulberry to phytoplasma [59]. Scharte *et al.* suggested that in order to initiate respiration and other processes that are required for the plant’s defense against the pathogen, photosynthesis must be switched off [60].

### Induction of Symptom Expression Proteins upon Phytoplasma Infection

This group encompasses genes involved in symptom expression. The rapid activation of a multitude of defense reactions, including regulation of the senescence-associated proteins and auxin-related proteins, initiates disease resistance in plant–pathogen interactions. [61]. Down-regulation of the senescence-associated proteins indicates a decrease in the senescence phenomenon in infected plants, which is in agreement with the fact that the chlorosis and mortality (as senescence symptoms) are not observed in this host when infected by phytoplasma [62]. As in the current study, the heat shock 70 kDa protein (encoded by m. 35228) involved in endocytosis (map 04144), a autophagy-related protein (encoded by m. 35982) involved in regulation of autophagy (map 04140), and two RING finger and CHY zinc finger domain-containing proteins (encoded by m. 46718 and m. 46722, respectively) involved in ubiquitin-mediated proteolysis (map 04120) are found to be differentially expressed. The under-expression of these genes is probably due to a low concentration either of ROS or to the blockage of nutrient transport. The differential regulation of auxin efflux carrier 5NG4 encoded by m. 21648 was detected in symptomatic parts and caused auxin accumulation. The auxins, as senescence inhibiting hormones [63], have been implicated in growth, morphology [64], and also in apical dominance [65]. Thus, under-expression of an auxin-transport related gene within PTFI plants could inhibit apical dominance and induce phytoplasma symptoms as was recently shown by the down-regulation of two auxin efflux carrier proteins in transgenic plants presenting proliferation symptoms [65]. The deregulation of genes in the symptomatic parts of plants induces the appearance of the specific proliferation symptoms in Paulownia.

### Differentially Expressed Genes Involved in Plant Defense upon Phytoplasma Infection

The most important group of the genes identified in this study are involved in the regulation of plant defense mechanisms. A pathogenesis-related protein encoded by m. 13687 is differentially expressed. A gene encoding a plant-pathogen interaction (map 04626) is selected. Metabolism of xenobiotics by cytochrome P450 occurs. The heat shock 70 kDa protein (m. 35228) in the MAPK signaling pathway (map 04010), antigen processing and presentation (map 04612), and the glutathione S-transferase (m. 31344) in the drug metabolism-cytochrome P450 pathway are in this group. H_2_O_2_ and other ROS play a role in phytoalexin synthesis. Reduction of H_2_O_2_, and consequently down-regulation of glutathione S-transferase, is responsible for high phytoplasma titers in the susceptible host. Thus, the plant defense mechanism is repressed and the higher susceptibility of the host and increased multiplication of the pathogen in PTFIs occurs. Auxin-induced protein 5NG4, previously mentioned in the symptom expression group, also interferes in plant defense regulation. Auxin homeostasis is one of the components participating in the regulation of the defense response [66]. Thus, deregulation of auxin-induced protein 5NG4 in this study suppresses the defense response pathways. All genes presented in the third group are notably involved in the suppression of the plant defense systems, possibly leading to high phytoplasma titers and then the death of the diseased host.

### Differentially Expressed Genes Involved in Secondary Metabolism upon Phytoplasma Infection

A gene (m. 32471) coding for ketol-acid reductoisomerase, a gene (m. 50337) coding for pyruvate kinase, and a gene (m. 15901) coding for phosphoribosylformyl glycinamidine cyclo-ligase, which are involved in the biosynthesis of secondary metabolites (map 01100), belong to this group. Various secondary metabolites have been found in defense responses to plant-pathogen interactions [61]. In the study of the ‘Bois noir’ phytoplasma induced grapevine, the transcripts of the genes encoding Ldox, which leads to the synthesis of anthocyanins, were up-regulated [55]. The RNA-seq analysis shows a modulation of the Paulownia gene expression in response to phytoplasma colonization and provides a first step towards the understanding of the phytoplasma-Paulownia interaction. To our knowledge, there has been no such extensive study at the transcriptome level regarding the interactions between Paulownia and phytoplasmas. In addition, the pathways proposed in the current study are presented for the first time as target pathways of phytoplasma infection in plants, except the effect of phytoplasma on photosynthesis. Further comparisons of the expression levels of identified genes in susceptible and resistant varieties or genotypes will enable the identification of molecular markers and genes involved in resistance or tolerance to the Paulownia proliferation disease.

## Conclusions

In the present study, we provided the first publicly available transcriptome resource for *P. tomentosa* × *P. fortunei*. More than 203,000 raw sequences were generated using Illumina RNA-seq. The analysis of these sequences has enriched our knowledge of *P. tomentosa* × *P. fortunei* and indicated the number of sequences that are significantly expressed during the process of PaWB disease resistance. These significantly expressed sequences have shed light on the biology of PaWB disease resistance in Paulownia. Finally, after BLAST searching, approximately 172,600 novel sequences remained unannotated, which represent potentially novel Paulownia genes. In conclusion, metabolic events are very active during the PaWB disease resistance stage. The sequenced unigenes in this study will be candidates for functional genomics and association studies in Paulownia species. This *P. tomentosa* × *P. fortunei* sequence resource, with its integrated information, offers an easy-to-use collection of information that will directly support further experimental studies and provide deeper knowledge on the transcriptome of *P. tomentosa* × *P. fortunei*.

This is believed to be the first transcriptome analysis of the response of *P. tomentosa* × *P. fortunei* to phytoplasma. The results provide evidence that phytoplasma causes significant changes in the levels of several transcripts and add to our understanding of the response of the Paulownia trees to phytoplasma infection.

There are several extensions of our work that will increase our understanding of plant responses to pathogens and may result in applications that enhance plant resistance. These extensions include the following: (1) Use of functional analysis to identify the unigenes that remain unidentified from the set of differentially expressed unigenes reported here. Identification of these unigenes may reveal further novel response pathways and the genes that control them. (2) Through the comparison of infected and healthy plants, several unigenes and pathways emerged as key participants in the disease response. These unigenes were shown to be involved in different processes, which included the oxidative stress response and photosynthesis. In addition, several pentatricopeptide repeat-containing protein unigenes were differentially expressed and these are possibly implicated in the plant’s defense against pathogens. These results can provide a framework for addressing new questions and designing experiments to elucidate the biology of plant–phytoplasma interactions. They might also serve as a basis to identify genes that can be targeted to enhance plant resistance or repress the growth and reproduction of the pathogen. However, further experimentation is required to elucidate the contributions of these genes in the susceptibility/resistance of *P. tomentosa* × *P. fortunei* trees to phytoplasma. In addition, the strategies to incorporate these genes into molecular breeding programs should be developed. (3) Examining highly responsive proteins coded by unigenes in other tissues and different time points. We are particularly interested in determining whether these unigenes or their corresponding transcripts can be used to detect stress responses at early stages. (4) It is important to understand whether the regulated unigenes reflect a direct effect of the interaction with the phytoplasma or a secondary effect of the development of symptoms. It is also interesting to determine whether the observed protein changes in response to pathogens are reflections of changes in gene expression or posttranslational modifications. Further studies are required to understand the roles of the regulated proteins.

## Supporting Information

Figure S1 **Distribution of unigene lengths in the transcriptome of** ***P. tomentosa*** **×** ***P. fortunei*** **.**
(TIF)

Figure S2 **Distribution of lengths of full length unigene in the transcriptome of** ***P. tomentosa*** **×** ***P. fortunei*** **.**
(TIF)

Figure S3 **KOG function analysis results of candidate defence genes.**
(TIF)

Figure S4 **GO function analysis results of candidate defence genes.**
(TIF)

Figure S5 **KEGG pathway analysis results of candidate defence genes.**
(TIF)

Table S1 **Annotation of differentially expressed genes.**
(XLSX)
