# Supplementary figures and images for: Discovery of Genes Related to Witches Broom Disease in Paulownia tomentosa × Paulownia fortunei by a De Novo Assembled Transcriptome
Source: PLoS One. 2013 Nov 21;8(11):e80238. doi: 10.1371/journal.pone.0080238 (PMC3836977; doi:10.1371/journal.pone.0080238)

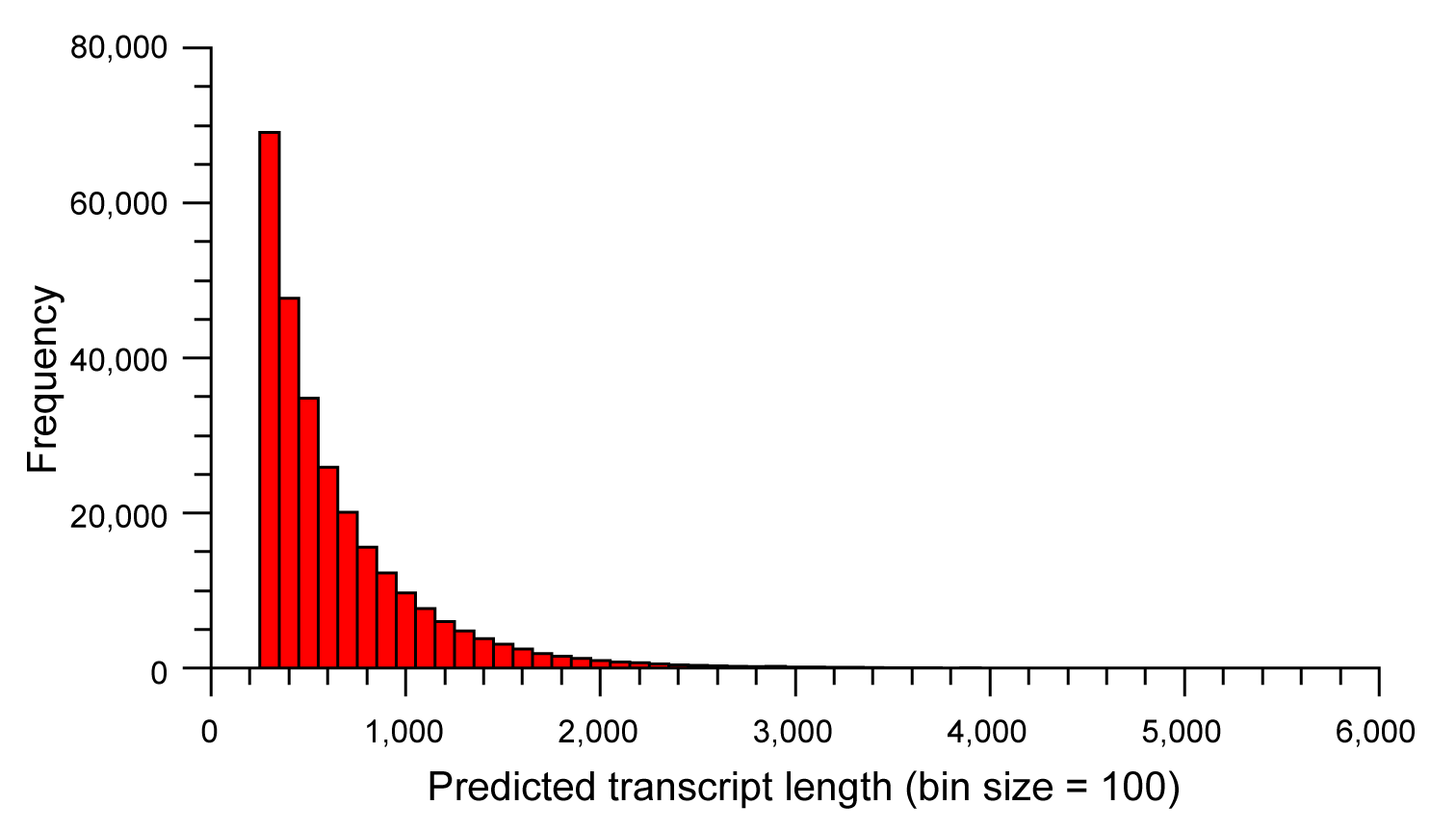

Supplement: Figure S1 — Distribution of unigene lengths in the transcriptome of P. tomentosa × P. fortunei . (TIF) [file pone.0080238.s001.tif]

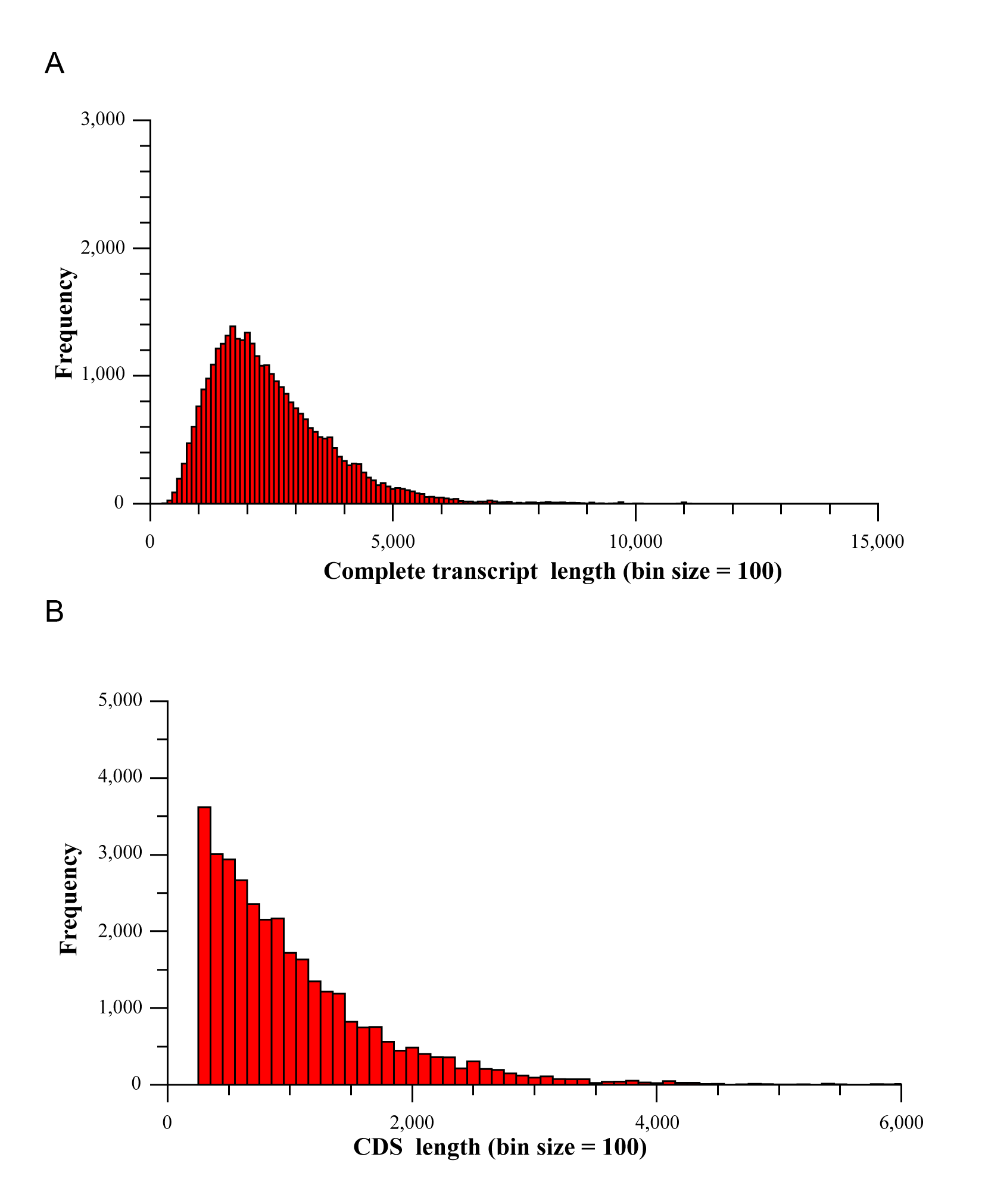

Supplement: Figure S2 — Distribution of lengths of full length unigene in the transcriptome of P. tomentosa × P. fortunei . (TIF) [file pone.0080238.s002.tif]

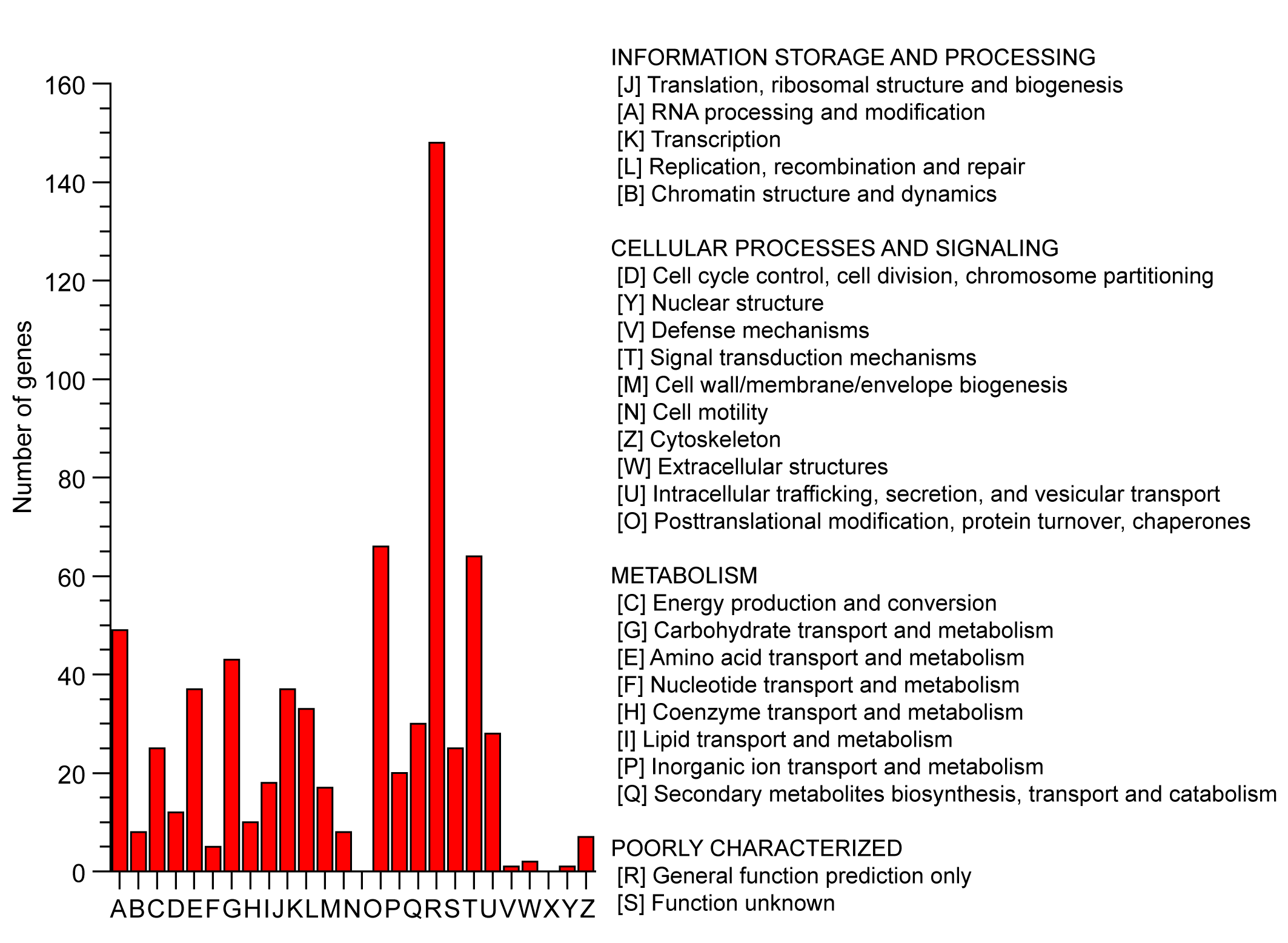

Supplement: Figure S3 — KOG function analysis results of candidate defence genes. (TIF) [file pone.0080238.s003.tif]

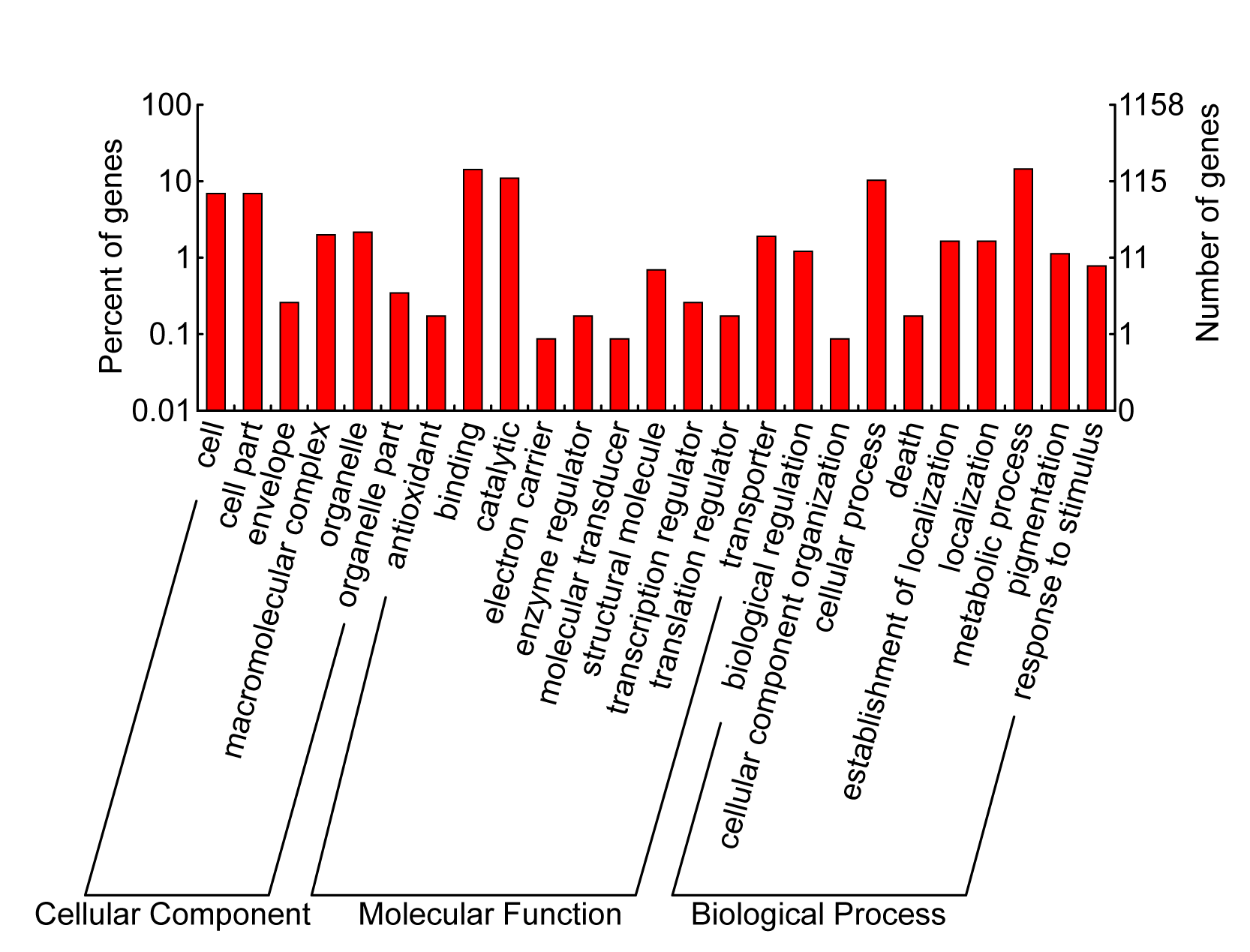

Supplement: Figure S4 — GO function analysis results of candidate defence genes. (TIF) [file pone.0080238.s004.tif]

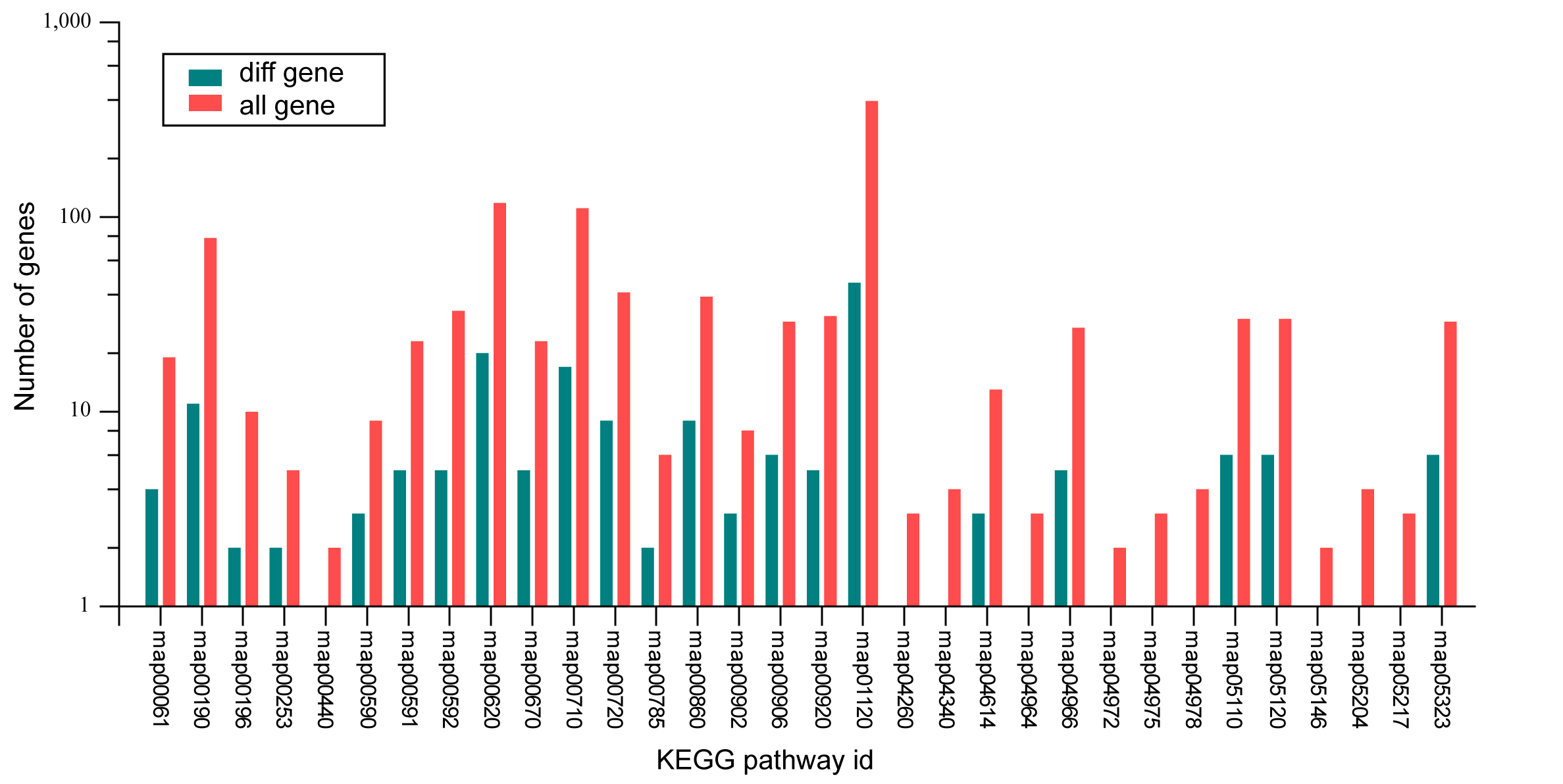

Supplement: Figure S5 — KEGG pathway analysis results of candidate defence genes. (TIF) [file pone.0080238.s005.tif]
